# Acute abdominal pain as the initial presenting symptom of pediatric Burkitt lymphoma: A case report and literature review

**DOI:** 10.1097/MD.0000000000041600

**Published:** 2025-02-21

**Authors:** Qing-Lin Tan, Qing Tang, Xiu-Qi Chen, Li Huang, Xiang Yun, Qing-Wen Shan

**Affiliations:** a Department of Pediatrics, The First Affiliated Hospital of Guangxi Medical University, Nanning, China; b Difficult and Critical Illness Center, Pediatric Clinical Medical Research Center of Guangxi, Nanning, China.

**Keywords:** acute abdominal pain, Burkitt lymphoma, children

## Abstract

**Rationale:**

Acute abdominal pain is a prevalent clinical symptom in pediatric patients, primarily attributed to pediatric acute abdomen. However, there are rare exceptional instances where neoplastic diseases may cause acute abdominal pain.

**Patient concerns:**

The present study encompasses a case of pediatric Burkitt lymphoma wherein acute abdominal pain manifested as the initial symptom, erroneously diagnosed as acute appendicitis.

**Diagnoses:**

The immunohistochemistry results of laparoscopic biopsy revealed Burkitt lymphoma, characterized by positive expression of CD20, CD79a, CD10, BCL-6, MUM1, and strong positive expression of CD38, c-myc, cyclinD1, and Ki-67 positive index >90%+.

**Interventions:**

The child underwent regular chemotherapy according to the SCCCG-BL/DLBCL-2017 regimen.

**Outcomes:**

During the 13-month follow-up period, the patient’s obtained complete remission by Positron emission tomography–computed tomography scan.

**Lessons:**

The objective of this study is to enhance the awareness of clinical pediatricians regarding cases of acute abdominal pain caused by lymphoma.

## 1. Introduction

The incidence of acute abdominal pain as a clinical symptom is significantly high in the pediatric population. Epidemiological surveys have revealed that the incidence of this condition varies across different age groups, ranging from 0.5% to 19%, with the peak occurrence observed between the ages of 4 and 12 years.^[1]^ Acute abdominal pain is also a very common chief complaint in the pediatric population, accounting for 5% to 10% of emergency department visits.^[2]^ The common causes of acute abdominal pain included gastroenteritis, appendicitis, intestinal invagination constipation, and urinary tract infection.^[3,4]^ Other less frequently encountered conditions that should also be taken into consideration include perforated ulcer, hemolytic uremic syndrome, ovarian/testicular torsion, and tumor.^[3]^ The diagnosis of acute abdominal pain caused by primary gastrointestinal tumors is challenging. This study presents a case of pediatric Burkitt lymphoma with acute abdominal pain as the initial symptom, initially misdiagnosed as acute appendicitis. The disease onset manifests as sudden abdominal pain, which gradually becomes insidious and poses challenges in diagnosis. Additionally, a literature review on cases of Burkitt lymphoma presenting with acute abdominal pain was conducted to enhance understanding of this condition.

## 2. Case presentation

**Figure 1.**
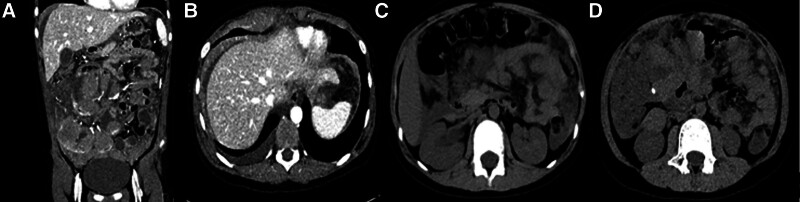
Abdominal CT: There were 2 oval high-density lesions in the small intestine in the abdomen (Se6/Img130, 193), the larger one was about 1.0 cm × 0.4 cm (B, D). There was no abnormal density shadow and abnormal enhancement focus in the remaining abdominal intestine. In the lower abdomen, the peritoneum, omentum and mesentery were thickened with blurred edges (A, C). Multiple lymph nodes were seen in the mesentery area, and the short diameter of the larger one was about 1.0 cm. CT = computerized tomography.

A 9-year-old boy presenting with abdominal pain as the primary symptom persisting for 1 week. The child exhibited paroxysmal and dull pain in the lower right abdomen, accompanied by abdominal distension and lumbago. The patient denied any history of emesis, diarrhea, arthralgia or arthritis, new-onset cutaneous eruption, dyspnea, palpitations, pyrexia, cough or any other discomfort. The patient was diagnosed with appendicitis at the local municipal people’s hospital due to the presence of an abnormal echogenic mass (hypoechoic mass, measuring approximately 50 × 16 mm) in the right lower abdomen as revealed by abdominal ultrasound. The patient is recommended to undergo an appendectomy. However, the patient’s guardian has declined the surgical procedure and opted for conservative treatment. After receiving cefixime treatment for 2 days, the patient experienced aggravated abdominal pain compared to their previous condition. The frequency of abdominal pain was higher than previously observed and there was progressive worsening of abdominal distension. Approximately 2 days later, the patient developed a fever with a peak temperature of 38.1 °C, accompanied by coughing and vomiting characterized by the presence of gastric contents without any evidence of coffee ground-like material. Concurrently, the patient presented with discolored yellowish and foul-smelling stool with 1 to 2 times per day. Subsequently, he was admitted to our hospital on the 10th day of the illness duration. The patient’s past history does not indicate any evidence of a diet lacking in hygiene or the absence of any familial occurrence of tumor history. The physical examination revealed normal vital signs, a slightly pallid complexion, lethargy, poor response, a comprehensive abdominal examination with tenderness on palpation and localized tenderness in the lower right abdomen. There was no rebound pain observed and an active mass was palpable. In addition, there were minor hepatosplenomegaly, and the findings on chest, cardiovascular, and central nervous system examinations were unremarkable. The blood routine examination revealed a white blood cell count of 9.29 × 10^9^/L, hemoglobin level of 126.0 g/L, platelet count of 303.0 × 10^9^/L, and neutrophil percentage of 0.677. High-sensitivity C-reactive protein was measured at 8.15 mg/L; interleukin-6 at 14.40 pg/mL; lactate dehydrogenase at 1469 U/L; erythrocyte sedimentation rate at 56 mm; Blood amylase and lipase tests yielded normal results. Purified protein derivative (PPD) tuberculin test and T cell spot test (T-Spot) were negative. The abdominal computerized tomography examination revealed thickening of the intestinal wall in the small intestine with decreased density and blurring of the surrounding fat space. Multiple lymph nodes were identified in the mesentery area (the largest measuring approximately 1.0 cm in short diameter), indicating inflammatory lesions as the underlying cause. Additionally, there was hydrops observed in the right kidney and upper/middle segment of the right ureter (Fig. 1).

**Figure 2.**
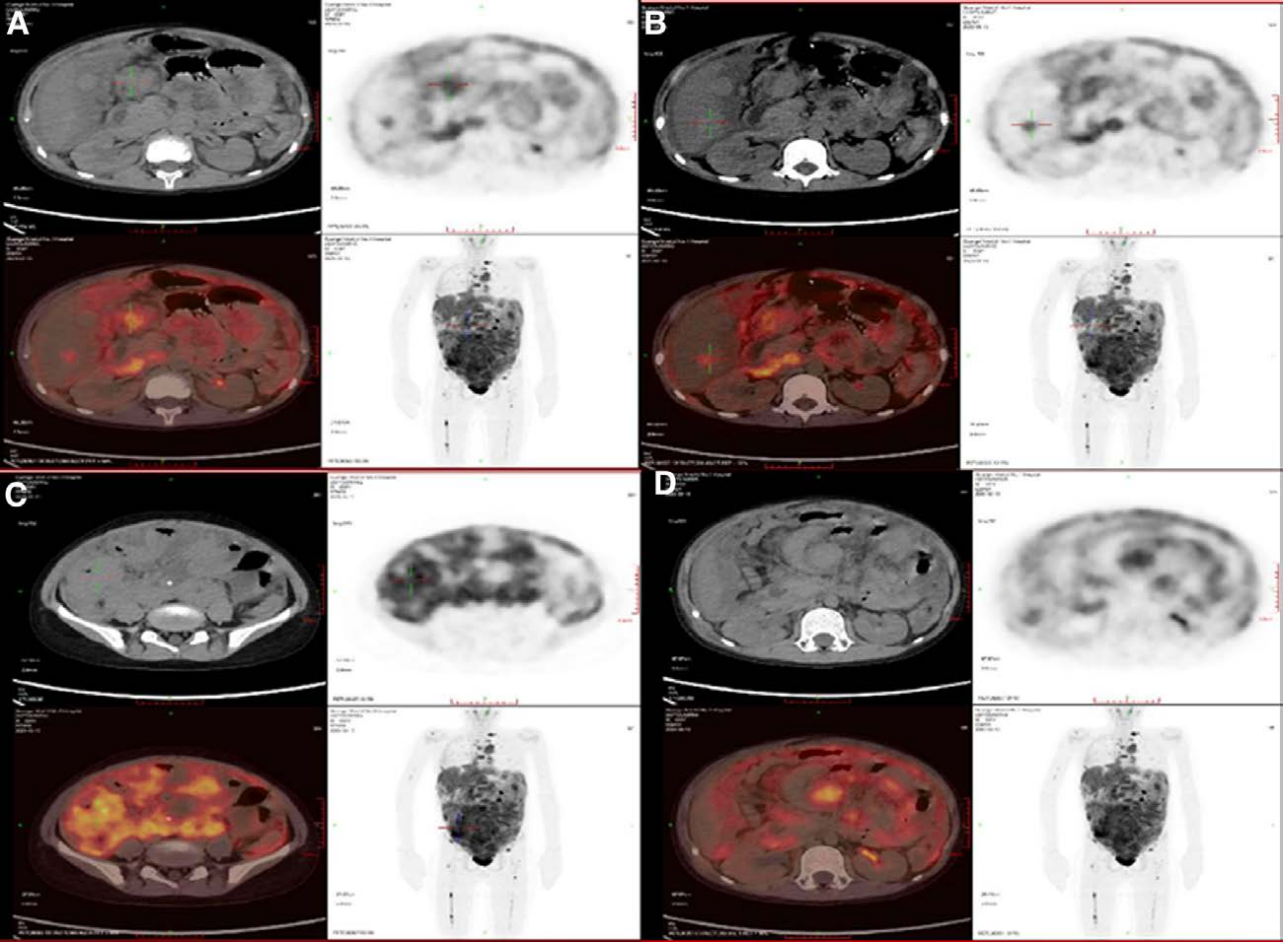
PET/CT confirmed diffuse sites of involvement localized in the abdomen. Increased glucose metabolism was observed in multiple locations, illustrations show involving the liver, peritoneum and pelvic cavity. PET/CT = Positron emission tomography–computed tomography.

After admission, the patient was administered a 7-day course of ceftazidime; however, there was no significant improvement observed in the patient’s condition. The patient continued to experience recurrent abdominal pain and significant weight loss, accompanied by headaches, vomiting, and the presence of ascites. The results of the diagnostic puncture for the abdominal cavity indicate: The routine ascites test reveals a Li Fanta test result of 2+ and a total count of nucleated cells at (3+/HP) × 10/L, with multilobed nuclear cells accounting for 90%. Ascites biochemistry shows an albumin level of 23.5 g/L and adenosine dehydrogenase activity at 39.1 U/L. Ascites picture examination showed a large number of naive cells. Lumbar puncture examination did not reveal any obvious abnormalities in routine and biochemical tests. The bone marrow puncture showed no characteristic pathological cells and neoplastic lesions were found. Positron emission tomography–computed tomography (PET–CT) examination indicates a high probability of lymphoma (lymphoma involvement in multiple tissues including numerous lymph nodes, right chest wall, liver, perihepatic capsule, peritoneum, omentum, abdominal and pelvic mesentery, bowel wall) (Fig. 2).

**Figure 3.**
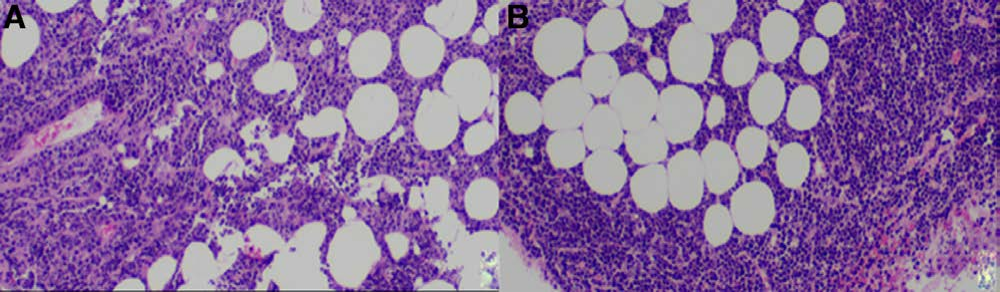
(A) (On the right side of abdominal tumor biopsy tissue): little blue round cell tumor. (B) (Mesenteric lymph node biopsy samples) microscopically, the huge medium large adipose tissue cells infiltrating lymphocytes samples, sample lymphocyte cell saw all over the sky star structure.

The possibility of lymphoma was considered base on the PET–CT, PPD tuberculin test and T-Spot results. Laparoscopic biopsy confirmed as Burkitt lymphoma (Fig. 3). The peritoneal omentum tissue revealed mass naive cells. Immunohistochemistry results showed positive expression of CD20, CD79a, CD10, BCL-6, MUM1, weakly positive expression of BCL-2, and strong positive expression of CD38, c-myc, cyclinD1, and Ki-67 positive index >90%+. Molecular pathology testing confirmed a positive MYC gene breakage test and MYC/IGH gene fusion. Following the diagnosis of Burkitt lymphoma, the child underwent regular chemotherapy according to the SCCCG-BL/DLBCL-2017 regimen. During the 13-month follow-up period, the patient’s obtained complete remission by PET–CT scan.

## 3. Discussion

The presence of acute abdominal pain is a prevalent symptom in pediatric emergencies, with the vast majority manifesting as benign lesions or acute abdomen. By employing laboratory examinations in conjunction with imaging and other auxiliary tests, most cases can be accurately diagnosed and exhibit a favorable prognosis. We reported a case of Burkitt lymphoma, with acute abdominal pain as the sole clinical manifestation. Due to tumor involvement in the ileocecal region, the patient presented with symptoms resembling “acute appendicitis,” which is commonly diagnosed in cases of acute abdomen.^[5]^ In this case, the initial lesion involved the ileocecal region, presenting typical symptoms indicative of appendicitis. Tumor invasion of the ileocecal region exhibited signs consistent with appendicitis accompanied by perforation and peritonitis on ultrasound examination; however, the patient’s blood routine and CRP infection indicators were within normal range, and there were no infection manifestations such as fever. We initially considered a differential diagnosis of intestinal tuberculosis, but PPD tuberculin test and T-Spot results were negative, and intestinal tuberculosis is usually accompanied by low-grade fever,^[6]^ so intestinal tuberculosis was excluded. In addition, the initial symptoms of Crohn disease (CD) can also present as abdominal pain, and some reports have suggested that gastrointestinal lymphoma can be easily misdiagnosed as CD.^[7]^ However, the patient’s disease progressed rapidly, the course of disease was short, and there were no significant extraintestinal symptoms, so consider the possibility of CD is low.

In addition, the common non-Hodgkin B cell lymphoma including BL and diffuse large B cell lymphoma,^[8]^ both and also need to differentiate between. The BL tumor cells were of medium size and showed a diffuse, unitary growth pattern. The tumor growth index and apoptosis index were high “Starry sky” is a common phenomenon. Some cases are difficult to distinguish from diffuse large B cell lymphoma. Immunohistochemically, the tumor cells were positive for ki-67, bcl-2, CD10, bcl-6, and CD38, but negative for bcl-2 and MUM-1. The same is true of immunohistochemistry in this case. In DLBCL, ki-67 expression was usually <90%, and bcl-2 was positive in about 50%, while CD10, bcl-6, MUM-1, and CD38 expression were different. Due to both the treatment and prognosis of different, thus the differential diagnosis is very important.

There is a paucity of literature reports on the definitive diagnosis of lymphoma presenting with acute abdominal pain. A literature review was conducted on Burkitt lymphoma cases presenting with acute abdominal pain as the initial symptom, and a total of 16 such cases were identified (Table 1).^[9–23]^ Among them, 3 cases exhibited concomitant fever, 3 cases had accompanying diarrhea and vomiting, 3 cases experienced weight loss, and 4 cases presented with constipation, 1 case presented with jaundice. Following the final diagnosis, localized lymphoma in the abdomen was observed in 15 cases, 1 case was found instances of distant metastasis. The demographics of this cohort were predominantly male, with the average age at presentation being 10.3 years old. The majority of cases clinically presented as classical acute appendicitis. The lesions were mostly located in appendix, and rare sites such as spine, stomach, ovaries could also be involved. And 37.5% (n = 6/16) of all cases mentioned immunohistochemical and fish. Only immunohistochemistry was performed in 25% (n = 4/16) of cases. An appendectomy (either open or laparoscopic) was the most commonly performed index procedure (n = 7/16, 43.7%). The follow-up period ranged from 2 months to 9 years. Most children can alleviate after treatment, only 2 cases died during the follow-up. Pathology examinations revealed that the typical heterotypic lymphocyte, is a gold standard for the diagnosis of Burkitt lymphoma. One thing is certain that almost all cases rely on postoperative pathological tissue to confirm the diagnosis. Endoscopic biopsy and pathological examination of surgical specimens are still the main basis for the diagnosis of pediatric Burkitt lymphoma.

**Table 1:** Literature review of childhood lymphoma associated with acute abdominal pain.

Author/year	Age/sex	Abdominal symptoms	Accompanying symptoms	Index procedure	Burkitt location	Pathological diagnosis	Duration of follow-up	Prognosis
Mimery/2019^[9]^	6/F	Focal peritonitis	Fever, poor appetite, and marked tenderness in the iliac fossa	Appendectomy	Appendix	Both	Not to mention	CR
Vrancx/2015^[10]^	17/M	Pain in the right lower quadrant	Ascites	Excisional biopsy	Terminal ileum	Both	2 mo	CR
Weledjil/2014^[11]^	13/F	Repeated dull pain in the right iliac fossa	–	Appendectomy	Appendix	Not available	8 yr	CR
Weledjil/2014^[11]^	18/F	Pain in right iliac fossa	–	Appendectomy	Cecum	Not available	9 yr	CR
Ryan/2013^[12]^	4/M	Persistent abdominal pain at night; an abdominal mass	Fever, non-bloody diarrhea, painful urination, and oliguria	Incision + drainage of purulent fluid	Appendix, sigmoid colon + mesentery, proximal rectum	Both	Not to mention	Not to mention
Grajo/2012^[13]^	7/M	Abdominal pain for 1 d	Vomiting and constipation	Ileocecectomy	Ileum and cecum	Both	8 mo	CR
Angotti et al/2012^[14]^	4/M	Recurrent spasmodic abdominal pain for approximately 1 mo	Anorexia, nausea, weight loss, vomiting, and constipation	Gastric biopsies	The stomach	Immunohistochemistry	Not to mention	CR
Wang/2010^[15]^	10/M	Intermittent periumbilical pain	Weight loss	Appendectomy; colonoscopy biopsy	Ascending colon	Immunohistochemistry	8 mo	Death
Bhardwaj/2010^[16]^	14/M	A 24-h history of tenderness in the right iliac fossa, with a soft mass palpable on physical examination	Diarrhea, fever	Right hemicolectomy	Terminal ileum	Not available	Not to mention	CR
Hafiz A. Yahya/2022^[17]^	15/M	Right abdominal pain	Constipation, tenesmus, dysuria, nausea, and vomiting	Appendectomy	Appendix	Not available	Lost follow-up	Not to mention
Fang/2003^[18]^	3/M	Persistent abdominal pain at night	Constipation, fecal incontinence, and weakness with tenderness in both lower limbs	Excisional biopsy of an epidural mass	Spine	Not available	16 mo	CR
Farahmandinia/2023^[19]^	2/M	Severe abdominal pain	Abdominal distension and vomiting	Appendectomy	Appendix, liver, kidney, and bone	Immunohistochemistry	Not to mention	CR
Lamrani/2024^[20]^	16/M	Epigastric pain	Jaundice, weight loss	Gastroscope biopsy	Pancreas and stomach	Immunohistochemistry	4 wk	Death
Persano/2023^[21]^	12/F	Right abdominal pain	Abdominal mass	Incisional biopsies from ovaryandomental nodules	Ovaries	Both	Not to mention	CR
Thompson/2018^[22]^	9/M	Right-sided lower abdominal pain	Anorexic, nausea, and vomiting	End-to-end anastomosis and an appendicectomy	Ileum	Not available	5 mo	CR
Kulendran/2018^[23]^	15/M	Irretractable abdominal pain	Vomiting and anorexia	Excisional biopsy	Terminal ileum	Both	Not to mention	CR

The characteristic feature of Burkitt lymphoma is the presence of multiple large soft tissue masses, often accompanied by intestinal stenosis.^[24]^ While complete obstruction is rare, some cases may exhibit incomplete intestinal obstruction. In the past, gastrointestinal lymphoma has typically presented with obvious enlarged primary lesions. However, in this study, the case exhibited abdominal pain and abdominal lymph nodes without significant lymph node enlargement on computerized tomography examination. The largest lymph node measured <2.5 cm, which may easily be overlooked in clinical practice. The patient underwent PET–CT examination, which prompted further confirmation of the diagnosis through surgical pathological biopsy.

The most prevalent form of non-Hodgkin lymphoma in children is Burkitt lymphoma, accounting for 30% to 50% of pediatric cases.^[25–27]^ The World Health Organization classifies Burkitt lymphoma into 3 subtypes based on clinical and biological characteristics: endemic, sporadic, and immunodeficiency-related Burkitt lymphoma.^[28]^ Among all non-Hodgkin lymphomas, gastrointestinal lymphoma accounts for 5% to 10% of cases.^[29]^ The majority of these cases arise in the stomach (up to 65% of all gastrointestinal lymphomas), followed by the small bowel (20%–30%), with the rest arising in the colon and rectum.^[29–32]^ The involvement of multiple organs throughout the body is a characteristic feature of Burkitt lymphoma, and sporadic Burkitt lymphoma-associated intestinal lymphoma primarily originates from the submucosal lymphoid tissue in the intestinal wall, often affecting the ileocecal region. About 31% of patients present with a palpable abdominal mass, some of which may manifest as unexplained abdominal pain, while others can present as intractable vomiting, intussusception, constipation, intestinal obstruction, and malignant ascites accompanied by systemic symptoms such as fever, night sweats, angular. In severe cases, it may even lead to intestinal perforation and death due to septic shock.^[33,34]^

In summary, this article presents a case study of Burkitt lymphoma in pediatric patients presenting with acute abdominal pain initially misdiagnosed as acute appendicitis. However, when the etiology of acute abdominal pain remains unclear, accurate diagnosis becomes challenging and consideration should be given to the possibility of lymphoma. It is imperative for clinical pediatricians to heighten their vigilance towards cases of acute abdominal pain associated with lymphoma. Gastrointestinal lymphoma is a rare but life-threatening disease that requires prompt diagnosis and appropriate management to reduce its high morbidity and mortality. And consider histopathological examination of all resected specimens of gastrointestinal-related tissue.

## Author contributions

**Conceptualization:** Qing-Lin Tan.

**Data curation:** Qing-Lin Tan.

**Formal analysis:** Qing-Lin Tan.

**Funding acquisition:** Xiu-Qi Chen, Qing-Wen Shan.

**Investigation:** Qing Tang, Li Huang, Xiang Yun.

**Methodology:** Qing Tang, Xiu-Qi Chen, Li Huang, Xiang Yun.

**Supervision:** Xiu-Qi Chen, Qing-Wen Shan.

**Writing – original draft:** Qing-Lin Tan.

**Writing – review & editing:** Qing Tang, Xiu-Qi Chen, Li Huang, Xiang Yun, Qing-Wen Shan.
